# Blood coagulation and the risk of atherothrombosis: a complex relationship

**DOI:** 10.1186/1477-9560-2-12

**Published:** 2004-12-01

**Authors:** Henri MH Spronk, Danielle van der Voort, Hugo ten Cate

**Affiliations:** 1 Department of Internal Medicine, University Maastricht, Maastricht, The Netherlands

## Abstract

The principles of Virchov's triad appear to be operational in atherothrombosis or arterial thrombosis: local flow changes and particularly vacular wall damage are the main pathophysiological elements. Furthermore, alterations in arterial blood composition are also involved although the specific role and importance of blood coagulation is an ongoing matter of debate. In this review we provide support for the hypothesis that activated blood coagulation is an essential determinant of the risk of atherothrombotic complications. We distinguish two phases in atherosclerosis: In the first phase, atherosclerosis develops under influence of "classical" risk factors, i.e. both genetic and acquired forces. While fibrinogen/fibrin molecules participate in early plaque lesions, increased activity of systemic coagulation is of no major influence on the risk of arterial thrombosis, except in rare cases where a number of specific procoagulant forces collide. Despite the presence of tissue factor – factor VII complex it is unlikely that all fibrin in the atherosclerotic plaque is the direct result from local clotting activity. The dominant effect of coagulation in this phase is anticoagulant, i.e. thrombin enhances protein C activation through its binding to endothelial thrombomodulin.

The second phase is characterized by advancing atherosclerosis, with greater impact of inflammation as indicated by an elevated level of plasma C-reactive protein, the result of increased production influenced by interleukin-6. Inflammation overwhelms protective anticoagulant forces, which in itself may have become less efficient due to down regulation of thrombomodulin and endothelial cell protein C receptor (EPCR) expression. In this phase, the inflammatory drive leads to recurrent induction of tissue factor and assembly of catalytic complexes on aggregated cells and on microparticles, maintaining a certain level of thrombin production and fibrin formation. In advanced atherosclerosis systemic and vascular wall driven coagulation becomes more important and elevated levels of D-dimer fragments should be interpreted as markers of this hypercoagulability.

## Background

The blood coagulation system comprises three basic elements: platelet adhesion, activation and aggregation, fibrin formation, and fibrinolysis. These elements interact with each other and with the blood vessel wall and under physiological conditions blood flow to tissues is unimpaired by clotting [1]. Under pathophysiological conditions, blood coagulation gets activated along the principles outlined by Virchov, which indicate that thrombosis (the formation of an intraluminal blood clot) always occurs through the interaction of three components: an altered vessel wall, an impaired or changed pattern of blood flow and an altered blood composition. The principles of Virchov's triad appear to be operational in each different type of thrombosis [2,3].

In *venous thrombosis *of the lower limbs, stasis, local inflammation on activated vascular endothelial cells induced by adhering leukocytes and platelets and in some cases direct vascular damage, promotes local thrombus formation. In a first episode of venous thrombosis the pre-existing composition of the blood is particularly important where congenital and acquired hypercoagulable factors such as factor V Leiden mutation and oral contraceptives, respectively, act in concert to accelerate clotting [4].

In *disseminated intravascular coagulation (DIC)*, widespread fibrin formation is the result of systemic inflammatory changes that induce cellular tissue factor dependent activated blood coagulation as well as local alterations in microcirculatory flow and enhanced activity and permeability of capillary endothelial cells [5]. Again, DIC follows Virchov's principles, i.e. interactions among all three elements occur which are all relevant determinants of outcome.

In *arterial thrombosis*, local flow changes and particularly vascular wall damage are the main pathophysiological elements. Alterations in composition of the arterial blood are also involved but the specific role and importance of blood coagulation is an ongoing matter of debate [6,7]. While numerous studies have shown increased activity of the blood coagulation system in patients at risk of arterial thrombotic complications, Tracy concludes on the basis of genetic studies that there is no "compelling argument supporting the importance of a preexisting hypercoagulable state as a major risk factor for atherothrombotic disease" [8]. In a recent debate, Reitsma points out that in the context of atherosclerosis a hypercoagulable state is of minor importance for the risk of thrombosis and high levels of coagulation factors such as factor VIII are risk indicators rather than causal factors [6]. On the other hand, in the same debate Grant argues on the basis of biochemical, clinical and philosophical arguments that hypercoagulability is indeed an issue of importance in arterial thrombosis, illustrated on the basis of several observations in patients with diabetes and insulin resistance [7].

In spite of the apparent controversies regarding this topic, observational studies focused on activity of coagulation and fibrinolysis in patients with arterial vascular disease continue to be published. As an example of a "clotting" marker, measurement of fibrin D-dimer fragments by one of many commercial assays, has been implicated as a risk indicator since more than 15 years, in a range of patient studies related to severity of atherosclerosis and/or risk of (recurrent) thrombotic complications [9-25]. In general, these studies indicate that D-dimer, similar to C-reactive protein (CRP), is a moderate but consistent and independent marker of risk of cardiovascular disease, both in population studies and in patients at risk [22,24,26]. Given the actual debate on the relevance of coagulation in arterial vascular disease it is timely to consider whether D-dimer should be regarded a risk marker (or *bystander*), or a marker of a *causal process*, i.e. hypercoagulability. More specifically, the question remains whether hypercoagulability, here defined as an increased potential to produce fibrin in plasma (indicated by elevated thrombin production, fibrin production or both), as compared to individuals of similar age and sex, should be seen as a cause or merely consequence of atherosclerosis and thrombosis.

## Thrombogenicity and atherosclerosis

In the majority of patients atherothrombotic complications develop on the basis of atherosclerosis in one or more coronary, cerebrovascular or peripheral arteries [27]. Atherosclerosis, a multifactorial disease, is the consequence of many years of exposure to atherogenic influences that lead already at young age to early lesions, or so-called "fatty streaks". Under influence of age- and sex-related factors these early lesions advance and this process is accelerated by genetic determinants (such as related to lipid and glucose metabolism and blood pressure) and environmental influences, including smoking and diet [28,29]. Arterial thrombi form in the course of progression to complex lesions, where the combination of vascular remodeling, erosion of the vessel luminal surface or frank rupture of plaques triggers the blood coagulation system. Central to this process is chronic inflammation and proteolysis culminating in plaque damage and exposure to luminal blood flow [27,30]. In addition, angiogenesis related neovessels are prone to rupture resulting in increased intra-plaque hemorrhage [31].

Activation of blood coagulation occurs primary through interaction of platelets, vessel wall and plasma proteins (so-called primary haemostasis). When injury to the blood vessel wall causes disruption of its endothelial layer, the underlying extracellular matrix is exposed. In this matrix, both von Willebrand factor (vWF) and collagen are present and after exposure, they will bind to specific receptors, glycoproteins (GP), present on the platelets. Dependent on the flow within the vessel other glycoproteins are involved in the adhesion of the platelets to the vessel wall. During low shear stress, GP Ia-IIa, GP VI and GP IV are the primary receptors for collagen, and during high shear forces, the primary indirect receptor for collagen is GP Ib-IX-V in a vWF dependent interaction. After adhesion of the platelets, they become deformed due to cytoskeletal changes, thereby exposing activated integrins and secreting ADP, serotonin etc. One of the integrins, GP IIb-IIIa, binds vWF (in high shear areas) or fibrinogen (in low shear areas) to mediate platelet aggregation under shear conditions. Also other platelet receptors and lipid products i.e. arachidonic acid, contribute to platelet aggregation. In this review however we will focus on secondary haemostasis, in which the interaction between circulating factor VII(a) to tissue factor, exposed by the damaged vessel wall, leads to activation of the coagulation cascade.

**Figure 1:**
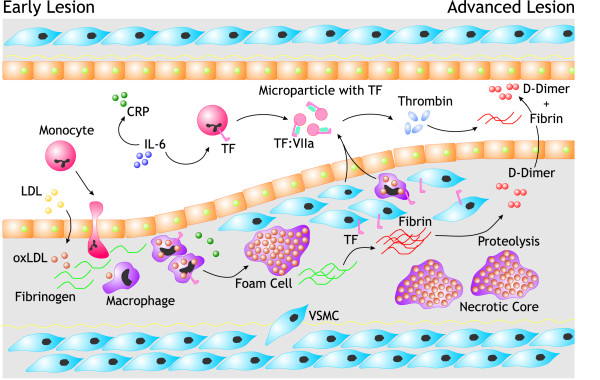
The initiation of an atherosclerotic lesion is characterized by retention of LDL and subsequent oxidative modification (oxLDL) within the matrix of the vascular intima. Stimulation of the overlying endothelial cells by oxLDL recruits monocytes from the circulation to the vessel wall. Differentiation of monocytes into macrophages and scavenger receptor mediated uptake of oxLDL aggregates results in the formation of foam-cells. Upon stimulation vascular smooth muscle cells (VSMC) migrate and proliferate. Tissue factor is expressed on macrophages and VSMCs within the advanced lesion and is likely to be involved in the conversion of accumulated fibrinogen into fibrin, although fibrin polymerization can be facilitated by other enzymes than thrombin. Furthermore, VSMCs and macrophage derived apoptotic bodies exposing TF probably contribute in thrombin formation. Considering atherosclerosis as a chronic inflammation, the inflammatory drive leads to IL-6 induced TF expression of circulating monocytes and the formation of microparticle exposing TF in the circulation, maintaining a certain level of thrombin production and fibrin formation. Increased circulating D-dimer levels are thus the result of fibrin proteolysis in both circulation and the advanced atherosclerotic lesion.

Several studies have shown that tissue factor is a prominent component of plaque lesions where it is localized in the outer membranes of infiltrating macrophages/foam cells and smooth muscle cells as well as on apoptotic cells and cell bodies (Figure 1) [30]. Unstable plaques contain most potent tissue factor activity; in addition, tissue factor-rich microparticles are being shed from activated and apoptotic cells and may contribute to acute thrombotic occlusion, particularly in the downstream microcirculation [30,31]. Formation of the tissue factor-factor VII(a) complex drives the intrinsic pathway of coagulation to form thrombin and fibrin. Platelet adhesion and activation, and interactions with leukocytes, accelerate the process of thrombin formation providing catalytic surfaces, expressing tissue factor and yielding coagulation proteases such as factor XIa that amplify thrombin generation [32-35]. According to Virchov's postulate acute arterial thrombosis occurs due to interaction among a damaged atherosclerotic vessel wall, an altered blood flow due to changes in shear stress related to atherosclerosis and blood elements, i.e. cells and coagulation proteins [3]. Whether the state of activity of the blood coagulation system (in other words "high risk blood") is really altered *prior to *thrombosis is the principal issue of controversy.

## The contribution of blood coagulation to atherosclerosis: the role of fibrin/fibrinogen and its split products

The involvement of coagulation in the pathological substrate of atherosclerosis is beyond dispute. For many years pathologists have noted the abundant presence of fibrin in advanced atherosclerosis and this finding has fueled part of the debate on the relevance of fibrin or fibrinogen for vessel wall lesions. Rokitansky and later Duguid proposed the encrustation theory as concept for the role of fibrin in atherosclerosis (reviewed in [36]). In this concept thrombosis was considered an etiological factor of importance in atherosclerosis, which was probably based on the presence of the end product of clotting, fibrin. Later work confirmed that fibrin is indeed an abundant protein in the arterial vessel wall, but not confined to atherosclerotic lesions. Schwartz and colleagues demonstrated that fibrin was also present (although at lower ratios of fibrin: fibrin/fibrinogen) in the non-sclerotic regions of the carotid artery where it did not co-localize with tissue factor in about 50% of the sections studied [37]. Thus, it seems unlikely that all fibrin in the vessel wall is the direct result from local clotting activation; alternatively, inflammatory influences that are characteristic of atherosclerosis [27] activate the coagulation system and also stimulate the transfer of fibrinogen and fibrin molecules to the intima where fibrin can be polymerized also by other enzymes than thrombin [38].

Autopsy data have indicated that fibrinogen accumulation in the vessel wall may be an *early *event in atherosclerosis, i.e. a small amount of fibrinogen in a thickened intima was demonstrated in a 4 year old boy [36]. The deposition of fibrinogen was apparently associated with the presence of LDL in the vessel wall and was related to age and intimal thickening. These authors suggested that intimal deposition of fibrin or fibrinogen preceded or facilitated LDL accumulation in the arterial vessel wall. Direct evidence for such a function of fibrin or fibrinogen, however, is still lacking. Fibrinogen knockout mice against an apoE^-/- ^background did not have fewer arterial lesions ranging from early lesions to complex fibrous plaques, suggesting that fibrinogen is not an essential molecule for atherosclerosis [39]. However, a later study demonstrated that fibrinogen was an important mediator of atherogenesis in apo(a) transgenic mice where the accumulation of apo(a) in the vessel wall and average lesion area were markedly attenuated in the fibrinogen^-/- ^x apo(a) crossbred animals [40].

The specific effect of fibrin and its split products in the vessel wall has also been studied. In general it appears that with increasing complexity of lesions there is an increase in the presence of intimal fibrinogen/fibrin and threads of fibrin, as well as an accumulation of various split products that may be involved in atherogenesis. The effect of fibrin and its split products on smooth muscle cells may be such that fibrin stimulates proliferation, while split products inhibit this process. Fibrin cleavage products may be detrimental for endothelial cell function, increasing permeability and promoting endothelial cell migration [36,41,42]. Degradation products also enhance chemotaxis of smooth muscle cells and monocytes. Extracellular accumulation of fibrin(ogen) on monocytes stimulates cholesterol transfer from platelets to monocytes/macrophages and each of these mechanisms may be relevant to the development of atherosclerosis. In addition, D-dimer fragments induce Il-6 production by monocytes in vitro [41,42].

The complex interactions of plasminogen, plasmin and its inhibitor, with regard to vessel wall function and remodeling, have recently been reviewed and its discussion is beyond the scope of this paper [43]. However, a few points need to be addressed. Plasmin, produced by activation of plasminogen, is the crucial enzyme in fibrin degradation and generation of split products. Deficiency of plasminogen in mice (Plg^-/-^) results in markedly discrepant effects on atherosclerosis. While Plg^-/- ^mice with an apoE^-/- ^background showed an accelerated development and progression of intimal lesions, Plg^-/- ^mice were protected against atherosclerosis in association with transplantation (reviewed in [43]). The origin of such apparently conflicting effects may lie in a dominant effect of the absence of plasminogen on lipid metabolism, including markedly lower HDL levels in the knockout mice in the first experiment, while an effect on leukocyte transport and migration was the major effect in the transplant experiments. Hence, as Plow et al conclude, "it may be the importance of the cellular migration as a rate-determining step that establishes the influence of plasminogen in either atherosclerosis or restenosis".

Accumulating fibrin that polymerizes in the vessel wall triggers fibrinolysis. Fibrinolytic enzymes tissue plasminogen activating factor and urokinase plasminogen activating factor (tPA and uPA, respectively) are present in intima and are secreted by endothelial cells and likely play an important role in vascular remodeling [42-44]. However, their intrinsic capacity to generate plasmin cleaving fibrin may also contribute to increased local fibrinolysis. In addition, the main inhibitor of fibrinolysis plasminogen activator inhibitor-1 (PAI-1) is also more abundantly expressed in tissues of patients with atherosclerosis. Under influence of inflammation, vascular endothelium may produce increased amounts of PAI-1 that might inhibit fibrin cleavage. However, it is questionable to what extend endothelial cells contribute to PAI-1 production in patients with atherosclerosis, since at least in patients with the metabolic syndrome, adipocytes and hepatocytes are more prominent sites of PAI-1 synthesis in relation to plasma PAI-1 [45].

The net effect on fibrin cleavage and progression of atherosclerosis cannot be estimated. The above mentioned experiments with Plg^-/- ^mice give important clues regarding the range of mechanisms that are influenced. Effective fibrinolysis may be important in limiting fibrin accumulation and atherosclerosis in the initial phases. However, upon stronger inflammatory stimulation the effect on cell trafficking into the vessel wall becomes more dominant and the outcome may reverse such that impaired fibrinolysis may limit atherosclerosis. The latter would imply that high levels of PAI-1 may even be protective against atherosclerosis under certain conditions. Consequently, high concentrations of D-dimers, reflecting active fibrinolysis, may indeed be regarded as a sign of progressive atherosclerosis under inflammatory conditions.

All of these issues may have therapeutic consequences since several drugs that are routinely used in patients with atherosclerosis including statins, angiotensin converting enzyme inhibitors as well as angiotensin receptor blockers appear to influence the balance of coagulation and fibrinolysis, which may influence atherosclerosis on the long term by altering vascular properties [42].

## Clinical studies of increased intravascular fibrin as indicator of severity of atherosclerosis; studies in peripheral arterial disease

As mentioned above, a large number of clinical studies in different groups of patients with atherosclerotic disease have generally shown that increased levels of D-dimer fragments in plasma are associated with an increased risk of severe atherosclerosis and an increased risk of vascular complications. In population based studies the contributable risk of an increased D-dimer level is quite small but statistically significant. In specific cohorts of patients the risk association is more outspoken, but of course here selection bias may produce slightly stronger associations than may be found in "real life".

Before addressing specific study findings a few general observations deserve attention.

First, strong associations between age and sex on the one hand, and D-dimer levels on the other hand, are noted [9,11,42]. D-dimer levels increase with age, are higher in women and may be influenced by a number of additional factors that differ per study.

Second, D-dimer levels are oftentimes associated with markers of inflammation, i.e. CRP and Il-6 [19,22,24,25,42,46]. In addition, D-dimers often correlate with fibrinogen levels, which may be related to inflammation, but fibrinogen is also the substrate for fibrin, thus a more straightforward substrate-enzyme-cleavage product relation may also play a significant role.

Before addressing the mechanisms we will consider D-dimer as an independent entity, i.e. a marker of disease severity. The most striking associations with clinical disease come from patients with peripheral artery disease (PAD), a reflection of systemic and advanced atherosclerosis in the majority of individuals. The total risk of clinical complications or mortality reaches figures of up to 25% annually in patients with PAD (48). In patients with PAD, elevated D-dimer levels are independent predictors of complications and are associated with severity of atherosclerosis [12-14,16,25]. Functionally, patients with PAD and highest D-dimers had the worst walking distance [23] and venous occlusion resulted in impaired fibrinolytic response in patients with PAD versus those without PAD [47]. Significant and independent associations between D-dimer and clinically relevant endpoints were also found in several studies in patients with PAD [15,18,25,48], in line with observations in other groups of patients with atherosclerotic manifestations.

Elevated levels of D-dimers are usually considered as a marker of increased clotting activity. This assumption is one of the key elements of the controversy regarding cause and consequence of hypercoagulability. Indeed, Herren et al observed increased levels of D-dimer in patients with PAD, correlating with severity of disease. They also noted an association between hypercoagulability (higher F1+2 and TAT) and occurrence of myocardial ischemia during exercise testing [13], suggesting a link between enhanced clotting activity, D-dimer levels and PAD. A similar link between activated clotting and higher D-dimer levels was also noted by van der Bom and colleagues showing that the association between D-dimers and severity of PAD was most apparent in those with highest thrombin cleavage fragment F1+2 and thrombin-antithrombin (TAT) levels [14]. However, a number of other studies in patients with atherosclerosis failed to reveal significant correlations between D-dimers and markers of thrombin generation [21,26]. This leads to the question whether D-dimer generation reflects hypercoagulability in blood, increased fibrin production and fibrinolysis in the arterial intima as part of advanced atherosclerosis, or an increased state of inflammation due to proteolytic cleavage of fibrin by neutrophilic enzymes such as elastase?

In spite of the substantial observational data, application of D-dimer assays or other risk factor measurements such as for CRP have not gained acceptance in individual patients with PAD or other cardiovascular disease yet. Thus, secondary prevention of complications is not guided by any laboratory assay, but limited to general recommendations such as the advice to stop smoking and the prescription of a platelet inhibiting drug [49]. Three reasons explain this lack of implementation: one is the substantial overlap in D-dimer (or CRP) values between normals and patients in general; second, the low specificity and three the lack of understanding the cause of the D-dimer production and its interpretation. In the context of this paper we will focus on the third reason and discuss the mechanisms that lead to elevated fibrin cleavage products in plasma in patients with atherosclerosis. Theoretically, there are different options to explain increased D-dimer levels in plasma. If D-dimers indicate increased *systemic clotting activity *then a specific anticoagulant intervention may theoretically be the preferred intervention. Clinical studies with anticoagulants in patients randomized or stratified on the basis of D-dimer levels have, however, not been carried out yet.

If however, D-dimers are a reflection of *severity *of atherosclerosis such an intervention may be inappropriate and potentially harmful because of the avoidable risk of bleeding and calcification of the arterial vessel wall upon long-term administration (at least with vitamin K antagonists). Alternatively, if D-dimer levels merely reflect *inflammation*, than therapy should preferably consist of anti-inflammatory agents including higher doses of aspirin, statins or ACE inhibitors. Thus, the interpretation of elevated D-dimer levels is quite important in order to guide decisions about individual therapy.

## Inflammation and fibrin formation in atherosclerosis

Atherosclerosis is a chronic inflammatory disease [27,28]. This widely accepted concept is based on a body of evidence from experimental and human observational studies. An indication of systemic inflammation is an elevated level of plasma CRP, the result of increased production influenced by Il-6. Several meta-analyses have established that CRP is an independent predictor of mortality in patients with atherosclerosis [50,51]; thus, a systemically activated inflammatory system is probably involved in its pathogenesis, i.e. progression and extension of atherosclerosis, as well as plaque rupture in advanced atherosclerosis.

Studies from the sepsis field including models of endotoxemia and sepsis in humans and primates, respectively, have shown that inflammatory stimulation leads to activation of blood coagulation [52,53]. Tissue factor synthesis is a rapid consequence of endotoxin infusion, which is a strong inflammatory stimulus, and this is followed by tissue factor expression on inflammatory cells and on microparticles, inducing thrombin and fibrin generation. Il-6 is a dominant cytokine in this process, but Il-1β and TNF-α are also involved. These experimental studies also exposed discordance in time of the coagulation activation steps, in which not a true cascade but a delayed and protracted course of activation occurred (1). If we consider atherosclerosis as a chronic (or recurrent) inflammatory condition, than the recurrent inflammatory drive leads to recurrent induction of tissue factor (with intermediate phases of hypo-responsiveness to stimulation) and assembly of catalytic complexes on aggregated cells and on microparticles, maintaining a certain level of thrombin production and fibrin formation [32,33]. The increased level of fibrinogen and fibrin monomers may enhance the uptake by the vessel wall of lipid-loaded particles and macrophages. In the vessel wall, further fibrin polymerization can occur due to local thrombin or other proteases activities.

In this concept, there is an increased generation of thrombin and fibrin in the blood circulation due to increased presence of inflammatory cytokines and proteins, but this does not necessarily lead to increased free thrombin in plasma. One should realize that coagulation enzymes that are generated associate with any available "scavenger", which can be an inhibitor such as antithrombin, but could also be a protease activated receptor (PAR) on platelets or endothelial cells [54-56]. Thus, a lack of rise of TAT at a moment when an elevated D-dimer level is noted cannot be interpreted as proof of a lack of increased thrombin production. Similarly, a lack of rise in F1+2 does not necessarily imply lack of thrombin production, because the F1+2 fragment can associate with cell membranes and little is known about the influence of microparticles. In addition, there are issues of sensitivity of commercial laboratory tests, i.e. the F1+2 assay is not a very sensitive tool in general, for monitoring activated coagulation. In fact, a D-dimer assay is the tool of choice for excluding (venous) thrombosis because of its superior sensitivity as compared to other tests for activated clotting [57]. We would propose that an increased production of thrombin in atherosclerosis is associated with an altered distribution of thrombin over the available binding sites leaving a greater procoagulant fraction that converts fibrinogen to fibrin. In advanced atherosclerosis a diminution in natural anticoagulant mechanisms including antithrombin (reduced expression of glycosaminoglycans at endothelium) and activated protein C (by down regulation of thrombomodulin) contributes to a higher level of procoagulant thrombin in the absence of increased TAT levels. Due to lack in sensitivity and maybe redistribution of F1+2 fragments binding to cell membranes the increased thrombin production is mostly undetectable by commercial F1+2 assays. Finally, discordances in peak levels of thrombin and fibrin production and cleavage may obscure any mechanistic associations.

The exact contribution of subendothelial fibrin formation and cleavage to D-dimer levels in blood cannot be estimated. While locally deposited tissue factor acts as a trigger of thrombin generation, it has not been shown that this is a source of ongoing subendothelial coagulation activity. The recent discovery of factor VII in plaque contents and the in vitro evidence for production of this Gla-protein by smooth muscle cells might form a basis for local thrombin production, but there is no indication yet that this might be quantitatively important as compared to hepatic production of factor VII [58].

The point to make is that high D-dimer levels in blood from patients with atherosclerosis should be primarily viewed as an indication of *systemic hypercoagulability*, a conclusion based on the above arguments and the experimental evidence indicating the intimate relationship between inflammation and coagulation [52].

## Inherited or acquired hypercoagulability and atherosclerosis?

Early studies from Rosendaal et al, suggested that thrombophilic traits including the prothrombin 20210 gene variant would be a risk factor for myocardial infarction in specific individuals such as heavy smoking young women [59]. These data led to speculations about the importance of inherited thrombophilia in arterial disease in general, but this association was refuted in a subsequent large study in young individuals [60]. Indeed, the prevailing opinion is that most known thrombophilic traits with an associated risk of venous thrombosis, do not influence the risk of arterial thrombosis [60,61]. This notion may lead to the erroneous assumption that a state of increased coagulation activity, irrespective the cause, would be of no significant influence for the risk to develop arterial thrombosis. In spite of discarding Rosendaal's data as being the result of bias due to lack of power and patient selection we should perhaps accept the possibility that in this specific group of individuals with an unhealthy lifestyle, hypercoagulable influences may have accumulated: smoking related inflammation (and tissue factor expression in arterial walls; [62]) in conjunction with estrogenic stimulation leading to an disproportionably high risk of arterial thrombosis even in the absence of overt atherosclerosis. In the majority of the population of young individuals (< 40 yrs) such scenarios do not play a major role and the risk of early atherosclerosis is influenced predominantly by "classical" risk factors. This concept matches with the observation that D-dimers levels are also no independent risk indicator in relatively healthy and younger populations including that of the Physicians Health Study [63].

In people of advanced age this situation may change considerably and the weight of risk factors may change over time. A study that specifically addressed this point is the Bruneck community study [64]. In this population study carotid artery atherosclerosis was monitored with duplex ultrasound, risk factors were recorded, baseline blood samples collected and individuals were prospectively followed in time. Conventional and clotting (candidate) risk factors were then linked to markers of disease. This study suggested a two-stage model of disease in which conventional risk factors such as dyslipidemia and smoking, influence early stages of atherosclerosis, while other factors including those linked to coagulation, influenced later stages of disease (Figure 1).

In advanced atherosclerosis the influence of coagulation may indeed be more prominent than in early stages, but it should be realized that acquired rather than genetically determined forces are involved. In this regard, the similarities between arterial thrombosis and the risk of recurrent venous thrombosis was used by Reitsma to make the point that inflammation is a key player under such conditions, reducing the influence that genetic thrombophilic background might inflict. On the other hand the same argument could be used to illustrate that indeed inflammation plays a more prominent role in advanced atherosclerosis where it more strongly drives the risk of thrombosis.

Let us consider this situation from the scope of venous thrombosis; this is not far edged because a recent study suggested similarities in risk factors between patients with previous venous thrombosis and atherosclerosis [65]. Recent studies clearly show that the risk of recurrent venous thrombosis depends on the one hand on persistent thrombus [66] as an inflammatory focus of disease as well as on persistent coagulation, i. e. elevated D-dimers, on the other hand [67]. Genetic influences such as factor V Leiden play no role of importance in the risk of recurrent venous thrombosis. In analogy with residual venous thrombus on the damaged venous vessel wall, advanced atherosclerosis represents a comparably damaged and inflammatory/thrombotic arterial vessel wall. Accordingly, similar plasma risk factors appear to be involved in recurrent venous thrombosis and arterial thrombosis (inflammatory markers and D-dimers).

## The "Bruneck" model of atherosclerosis and recommendations

The influence of blood coagulation on atherosclerosis follows a two stage model in which variants may occur under exceptional conditions. In general in the first phase, roughly covering the first four decades of life, atherosclerosis develops under influence of "classical" risk factors, including hypercholesterolemia and smoking, i.e. both genetic and acquired forces. While fibrinogen/fibrin molecules participate in early plaque lesions, increased activity of systemic coagulation is of no major influence on the risk of arterial thrombosis, except in rare cases where a number of specific procoagulant forces collide. The dominant effect of coagulation is anticoagulant, i.e. thrombin enhances protein C activation through its binding to endothelial thrombomodulin. Defects in the protein C mechanism may indeed precipitate arterial thrombosis, but only under highly thrombogenic conditions. Fibrinolysis limits fibrin accumulation in the intima and herewith progression of plaque lesions. At this stage elevated PAI-1 levels may diminish fibrinolysis and may stimulate plaque progression, which may explain that in a large Japanese study the PAI 4G/5G polymorphisms appeared to be a risk factor for myocardial infarction in women [68].

The second phase is characterized by advancing atherosclerosis, with greater impact of inflammation and increased infiltration of fibrin in the arterial vessel wall, enforcing pro-inflammatory effects. The extensive interactions between inflammation and coagulation enzymes and inhibitors (in much greater detail than discussed here) amplify the chain of events that determine the risk of atherothrombosis. Inflammation overwhelms protective anticoagulant forces, which in itself may have become less efficient due to down regulation of thrombomodulin (TM) and endothelial cell protein C receptor (EPCR) expression. In this phase, evidence of activated coagulation is measurable in peripheral blood reflecting both the extent of atherosclerotic burden and the systemic clotting tendency, which poses a direct risk of thrombotic complications. This point of view deviates from Tracy's viewpoint and provides a more constructive model for integrating coagulation in arterial disease.

We would also propose that D-dimer and other, novel assays such as for endogenous thrombin generation ("endogenous thrombin potential") [69] or for activated factor XII (suggested as a novel risk factor for cardiovascular disease [70]), be used to determine prospectively the risk of new atherothrombotic complications and to guide randomized intervention trials in patients stratified on the basis of such plasma markers.
